# Active Transposition of Insertion Sequences by Oxidative Stress in *Deinococcus geothermalis*

**DOI:** 10.3389/fmicb.2020.558747

**Published:** 2020-11-05

**Authors:** Chanjae Lee, Kyungsil Choo, Sung-Jae Lee

**Affiliations:** Department of Biology, Kyung Hee University, Seoul, South Korea

**Keywords:** *Deinococcus geothermalis*, transposition, insertion sequence, oxidative stress, LysR family regulator, OxyR, catalase

## Abstract

Radiation-resistant bacterium *Deinococcus geothermalis* has a total of 73 insertion sequences (ISs) in genomes, and some of them are actively transposed to other loci with replicative mode due to oxidative stress of hydrogen peroxide treatment. Here, we detected two transposition events in wild-type (WT) strain and LysR family member gene disrupted strain (Δ*dgeo*_2840). Similar to our previous report (Lee et al., 2019), phytoene desaturase (*dgeo*_0524), a key enzyme of carotenoid biosynthesis, was disrupted by the integration of IS element, thereby detected a single phenotypically non-pigmented colony in each WT and Δ*dgeo*_2840 strain. Two separate types of IS element have been integrated into non-pigmented clones: IS*Dge11* for WT and IS*Dge6* for Δ*dgeo*_2840 strain. Surprisingly, Δ*dgeo*_2840 mutant strain revealed higher resistance to oxidative stress than WT strain at late exponential growth phase. From the qRT-PCR analysis, OxyR (*dgeo*_1888) was highly up-regulated to 30-fold by oxidative stress through hydrogen peroxide treatment in both WT and Δ*dgeo*_2840 mutant strains. However, the oxidative stress response enzyme, catalase or superoxide dismutase, was not significantly induced by overexpressed OxyR. Thus, a putative LysR family regulator Dgeo_2840 controlled the expression of IS*Dge6* type transposase and the induction of OxyR under oxidative condition. There is LysR family DNA-binding protein dependent active transposition of specific type IS and the up-regulated OxyR has not positively controlled ROS scavenger enzymes in *D. geothermalis*.

## Introduction

Genus *Deinococcus* species can generally survive extreme and/or harmful conditions such as high stress of radiation, oxidative stress, desiccation, toxic substances, and starvation (Battista et al., 1999; Cox and Battista, 2005; Slade and Radman, 2011). For the survival strategy of the *Deinococcus* strain under various stressors, *Deinococcus radiodurans* has been well-studied for oxidative stress responses, and appears to coordinate in multiple ways, separated into five categories as described below: (1) DNA repair systems such as RDR regulon Rec, Ssb, Uvr, and Ddr proteins; (2) high efficiency of enzymatic reactions, such as catalase, peroxidase, and superoxide dismutase; (3) unique protective deinococcal proteins, such as Irr and Ppr, and DNA protection protein in stress condition such as Dps; (4) protective small molecules: pigment compound, such as carotenoids, metal ions, such as manganese, and other antioxidant systems including redox potential control by bacillithiol and cysteine residues; (5) stressors response regulators such OxyR, SoxRS, and RpoR (Loprasert et al., 1996; Battista et al., 1999; Hua et al., 2003; Makarova et al., 2007; Daly, 2009; Helmann, 2011; Slade and Radman, 2011; Agapov and Kulbachinskiy, 2015; Kim et al., 2017; Lim et al., 2019). Our research model organism, *Deinococcus geothermalis* has high radiation dose yielding 10% CFU survival, D_10_-10 kGy, and high intracellular Mn/Fe concentration ratio 0.46, shows reddish and/or orange-pigmented colony, and optimally grows at 45–50°C (Ferreira et al., 1997; Brim et al., 2003; Daly et al., 2004).

The LysR-type transcriptional regulator (LTTR) family is the most abundant group of transcriptional regulators that are highly preserved in prokaryotes. LTTRs may act as the most common type of positive regulators and/or also as some diverse negatively regulated genes and functions. Some LTTRs control regulons that form complex regulatory network by other genes. On the other hand, others regulate themselves (Schell, 1993; Maddocks and Oyston, 2008). The famous transcriptional regulator OxyR of LTTR main member positively controls a number of oxidative stress response related genes in *Escherichia coli*, such as catalase/hydroperoxidase I (*katG*), a non-specific DNA-binding protein (*dps*), alkyl hydroperoxide reductase (*ahpCF*), glutathione reductase (*gorA*), glutaredoxin 1 (*grxA*), and a small regulatory RNA (*oxyS*). In *D. radiodurans*, however, it represses two *dps* genes (Christman et al., 1989; Storz et al., 1990; Schell, 1993; Tao, 1997; Chiang and Schellhorn, 2012; Wei et al., 2012; Imlay, 2015). For example, strains carrying deletion of OxyR gene were unable to induce few genes in *Bacteroides fragilis*, and were sensitive to H_2_O_2_ or other oxidants, such as *tert*-butyl hydroperoxide in *Streptomyces avermitilis* (Rocha et al., 2000; Liu et al., 2016). However, Gram (+) bacterium *Corynebacterium glutamicum* revealed that when *oxyR* was deleted, the mutant strain showed increased resistance to H_2_O_2_ (Milse et al., 2014). The genome of *D. geothermalis* contains several LysR member regulators. In a previous work, quantitative RT-PCR analysis was performed on four LysR genes, including OxyR for oxidation and reduction states. OxyR was highly up-regulated on hydrogen peroxide treatment or the oxidized condition through cystine-importer disruption (Kim et al., 2017; Choo et al., 2020). Other LysR-member protein Dgeo_2840 was also induced in both early and late exponential growth phase and oxidative stress conditions (data not shown). In this study, Dgeo_2840 was selected to define physiological characteristics and built a disrupted mutant strain of *dgeo_*2840 gene (Δ*dgeo_*2840). During the oxidative stress resistance assay of Δ*dgeo_*2840 mutant strain comparing to the response of wild-type strain, we identified two colorless mutants from wild-type and Δ*dgeo_*2840 mutant strain. We hypothesized that IS transposition occurred in carotenoid biosynthesis pathway.

Bacterial inserted sequence (IS) is a simple transposable element consisting of a length less than 3,000 bp, with a single or two transposases and typical repeat sequences, such as terminal inverted repeat (TIR), and direct repeat (DR) sequences and is classified 30 family members followed criteria (Mahillon and Chandler, 1998; Siguier et al., 2015; Vandecraen et al., 2017; Makałowski et al., 2019). Following the transposition ability to break DNA strands and insert them more or less randomly into other loci of genomic DNA, two separate action modes, such as copy-and-paste and cut-and-paste transposition, result in the potential to dramatically destroy the genome contents (Mennecier et al., 2006; Pasternak et al., 2010; Siguier et al., 2014; Hickman and Dyda, 2016; Vandecraen et al., 2017). These IS elements distributed multiple copies with many different family types on genomes, and were easily defined through IS finding platforms (Sawyer et al., 1987; Siguier et al., 2006). For the *D. geothermalis* genome, there are 19 IS types in a total of 73 IS elements. The maximum number of copies of IS type is 15 copies of IS*Dge2* (ISfinder)^1^. In general, IS element is transposed to other sites in genomic DNA by the oxidative stress via reactive oxygen species (ROS) which is produced from H_2_O_2_ and γ-irradiation, high-temperature, and other DNA-damage toxic substances (Narumi et al., 1997; Ohtsubo et al., 2005; Pasternak et al., 2010). However, no individual transpositional action mechanism is clearly explained at all, because the key player transposase was controlled by multiple factors and phase points, such as transcriptional and translational control, and regulation at gene and protein network (Nagy and Chandler, 2004; Siguier et al., 2017). We have found in previous studies that certain ISs types, IS*Dge5* and IS*Dge7* actively transposed to other sites on genomic DNA under a special DNA-binding protein Dgeo_0257 disrupted state through oxidative stress treatment (Lee et al., 2019). IS*Dge7* type integrated on a key enzyme of carotenoid biosynthesis result in colorless phenotype. In addition, IS*Dge5* type was transposed into two other sites in genome of this non-pigment mutant (Lee et al., 2019). When carotenoid biosynthesis normally occurs, *D. geothermalis* strain is reddish in color. The carotenoids act as photooxidation protectors against damage from oxygen or ultra-violet or gamma-radiation. Carotenoids are produced as reactive oxygen species (ROS) scavengers in many non-phototrophic bacteria for cellular protection (Tian and Hua, 2010). Here, we observed two non-pigment mutants from each strain of the reddish wild-type (WT) and the mutant with *dgeo*_2840 gene destruction when oxidative stress was treated. We identified transposition loci in the carotenoid pathway, especially *dgeo*_0524 as phytoene desaturase, to determine the cause of the non-pigment phenotype. We believe that our study of whether special types of insertion sequences are induced and transposed into other loci in genomic DNA through extracellular and intracellular factors such as types of oxidative stressors and types of DNA-binding proteins will be an interesting and important question.

## Materials and Methods

### Strains, Culture Conditions, and Construction of the Mutant

The strain *D. geothermalis* DSM 11300^T^ was obtained from the Korean Agricultural Culture Collection (KACC 12208). *D. geothermalis* was cultured on TGY medium containing 1% tryptone, 0.5% yeast extract, and 0.1% glucose at 48°C. *Escherichia coli* DH5α was used as a competent cell for the transformation of recombinant DNA, and grown on Luria-Bertani medium at 37°C. The *dgeo_*2840 gene locus disrupted mutant strain was constructed by integration of a kanamycin-resistance cassette into a target gene through homologous recombination, following previous study (Lee et al., 2019). For the *dgeo_*2840 mutant strain, the homologous DNA sequence downstream of *dgeo_*2840, and also upstream region (roughly 1.0 kb), was amplified from genomic DNA using target-region primers, and purified using a PCR purification kit (Bioneer, South Korea). Firstly, the purified right border DNA fragments and plasmid pKatAPH3 were cleaved by *Xba*I*-Pst*I, and ligated into a plasmid (named pKR2840). Then to yield pKRL2840 as a left border DNA fragment ligation, the purified DNA fragments and plasmid pKR2840 were digested with *Kpn*I*-Sal*I, ligated, and propagated in *E. coli*. The final recombinant plasmid pKRL2840 was purified from *E. coli*, and transformed into *D. geothermalis* competent cell using a CaCl_2_-dependent technique described previously (Kim et al., 2017). The resulting transformed strain was named Δ*dgeo_*2840 mutant, and selected on TGY agar containing 8 μl/ml kanamycin for 2 days incubation at 48°C. The complementation assay of *dgeo*_2840 gene was performed following previous study (Choo et al., 2020).

#### Non-pigmented Mutants, Growth Property, and Viability Test on Oxidative Stress

The non-pigmented mutant strains were isolated from oxidative stress assays using hydrogen peroxide treatment. When Δ*dgeo_*2840 mutant strain was treated by 100 mM H_2_O_2_, a non-pigmented clone was selected that resulted in the mutant strain named Δ*dgeo_*2840*w*. From the 5th oxidative stress applied plate, an additional non-pigmented mutant came from wild-type strain that was treated with 50 mM H_2_O_2_ of serial trials, which was named WT*w*.

To evaluate the growth curve of the wild-type and Δ*dgeo_*2840 mutant strains of *D. geothermalis*, wild-type (WT), WT*w*, Δ*dgeo_*2840 mutant, and Δ*dgeo_*2840*w* were grown overnight in TGY broth at 48°C. Then the strains were inoculated to OD_600_ 0.06 in TGY broth, and continuous growth of the strains was monitored hourly.

To evaluate viability test on hydrogen peroxide treatment, the WT and Δ*dgeo_*2840 mutant of *D. geothermalis* were grown to an OD_600_ 2.0 for early exponential phase or OD_600_ 4.0 for late exponential phase in TGY broth at 48°C. The cells of identical OD_600_ 2.0 from each culture were exposed to hydrogen peroxide with 50, 80, and 100 mM of final concentration, and incubated continuously for 1 h. The stressed cells were serially diluted 10-fold in buffered saline from 10^0^ to 10^–5^. A 5 μl volume from each diluted suspension was spotted on the TGY agar plates, incubated at 48°C for 1 or 2 days, and analyzed by photography and colony counting.

#### Transcriptomic (RNA-Seq) Analysis

WT and the Δ*dgeo_*2840 mutant *D. geothermalis* strains were harvested at OD_600_ 4.0 in TGY broth at 48°C for RNA-Seq analysis. The total RNA was extracted by the RIDOEx reagent (GeneAll, South Korea). The extracted total RNA was purified using an RNeasy Mini Purification Kit (Qiagen, Germany) and RNase-Free DNase I Set (Qiagen, Germany). We commissioned *D. geothermalis* bacterial RNA-Seq, and data analysis was performed using the ExDEGA analysis tool of e-biogen Co. (South Korea). The data discussed in this study have been deposited in NCBI’s Gene Expression Omnibus (Edgar et al., 2002) and are accessible through GEO series accession number GSE151903.

#### Transposition Detection and Determination of Integration Loci

We designed target-gene-encompassing primer sets with similar melting temperatures for the detection of transposition loci, and performed PCR of four carotenoid biosynthesis involved enzyme genes, following our previous study (Lee et al., 2019), because the transposase-integrated sites were enlarged in the non-pigmented mutant, compared with WT and Δ*dgeo_*2840 mutant strains. From following PCR and agarose gel electrophoresis, the enlarged PCR products were selected, and then sequenced. The type of IS and transposase, integrated sites, and specialized DR and TIR sequences were determined. Transposition mode was also determined using PCR detection of distributed identical IS type genes in genomic DNA.

#### Quantitative Reverse Transcriptase (qRT)-PCR

To determine the expression levels of target transposase genes and oxidative stress related genes, we performed qRT-PCR, as previously reported (Kim et al., 2017). After cells were harvested at OD_600_ 4.0, cells were diluted to OD_600_ 2.0, and total RNAs were extracted using a phenol-based RNA extraction procedure. cDNAs were synthesized by PCR using reverse transcriptase (Qiagen, Germany), and the synthesized cDNAs were quantified by DeNovix (United States), normalized, and stored at −70°C, until real time PCR performance. Quantitative PCR was conducted using the RT-PCR machine (model CFX96^TM^ Optics Module, Bio-Rad, United States). Relative gene expression levels were calculated using the comparative threshold cycle (ΔΔ*C*_T_) method and normalized to the expressed level of the gene encoding glyceraldehyde-3-phosphate dehydrogenase (GAPDH), such as a control for stable expression level on H_2_O_2_ treatment (Livak and Schmittgen, 2001)^2^. The two-way ANOVA was used to test difference between the samples which were represented the means and standard deviations (SD) of three replicate experiments and it was considered to be significant at *p* < 0.05.

## Results

### Detection of Non-pigmented Clones From Wild-Type and Δ*dgeo*_2840 Mutant

**FIGURE 1:**
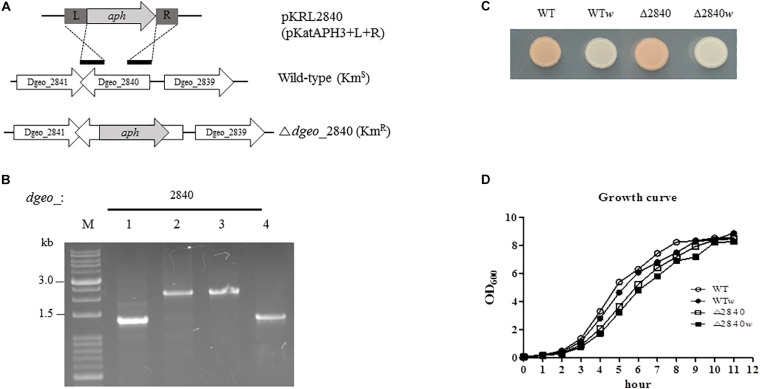
Construction of Δ*dgeo*_2840 mutant strain, phenotypic properties, and growth pattern. **(A)** Scheme of construction for the disruption of target gene *dgeo*_2840. “L” and “R” are left and right border, respectively, of *dgeo*_2840 gene for the recombination procedure. “*aph*” is a kanamycin resistant gene from pKatAPH cloning vector. **(B)** PCR amplification of genomic DNA from the wild-type and Δ*dgeo*_2840 mutant strains to confirm the insertion of *aph* gene. **(C)** The phenotypic color comparison between WT and Δ*dgeo*_2840 mutant parent strains and non-pigmented mutants. **(D)** Growth curves between parent strains and non-pigmented strains of WT and Δ*dgeo*_2840 mutant strains. Lanes: M, size marker; 1, WT; 2, mutant with Δ*dgeo*_2840; 3, Δ*dgeo*_2840*w*; 4, WT*w*. This datum indicated that Δ*dgeo*_2840*w* originated from Δ*dgeo*_2840 mutant, while WT*w* originated from wild-type strain.

To determine the functional role of the putative LysR family regulator Dgeo_2840, we constructed target gene disrupted mutant strain using homologous recombination procedure, and accurately detected using size enlargement of the target gene amplification and DNA sequencing (Figures 1A,B). In complementation assay, the shuttle vector pRADgro was used (Choo et al., 2020) and the recombinant DNA including intact *dgeo*_2840 gene was transformed into wild-type and Δ*dgeo*_2840 mutant strain. The empty vector and recombinant DNA were well-transformed into wild-type strain and selected on chloramphenicol contained medium (Supplementary Figure S1). Unfortunately, Δ*dgeo*_2840 mutant strain did not allow for the transfer of foreign DNA.

During the oxidative stress response assay in wild-type and Δ*dgeo*_2840 mutant strain, we isolated a non-pigment colony of WT treated by continuous culture with 50 mM H_2_O_2_. A single white colony, WT*w*, was acquired from the fifth trial. Another Δ*dgeo*_2840*w* colony came from the 100 mM H_2_O_2_ treatment on Δ*dgeo*_2840 mutant strain (Figure 1C). Both these non-pigmented clones were found to have slightly delayed grown by ca 10–15 min than their parent strains (Figure 1D). Δ*dgeo*_2840 mutant strain revealed an hour-delayed growth pattern as compared to WT, but reached the maximum growth density (Figure 1D). Thus, a member of LysR family Dgeo_2840 is not an essential gene.

**FIGURE 2:**
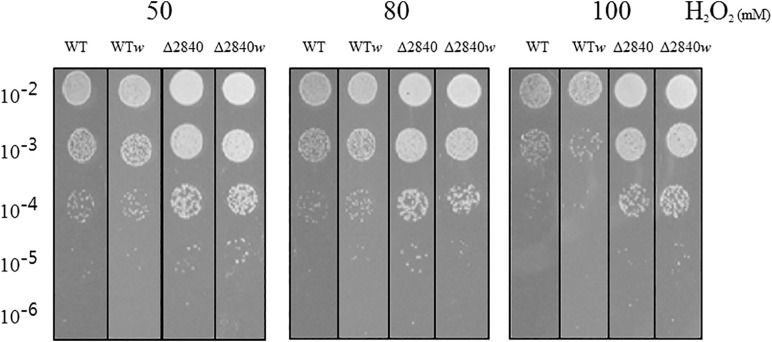
Viability test on oxidative stress. Resistance analysis among WT, WT*w*, Δ*dgeo*_2840, Δ*dgeo*_2840*w* at OD_600_ 4.0 on 50, 80, and 100 mM H_2_O_2_ conditions. After 1 h exposure, each lot of cells was serially diluted by 1/10, spotted 5 μl on TGY agar plate, and incubated at 48°C for overnight.

We performed a viability test to verify resistance to oxidative stress by H_2_O_2_. At early exponential growth phase, viability was not affected among tested strains (data not shown). Surprisingly, Δ*dgeo*_2840 mutants showed better viability than WT under oxidative stress with variable H_2_O_2_ concentration in OD_600_ 4.0 growth cells (Figure 2). When comparing red and white (non-pigmented) strains, the non-pigmented strain of WT was slightly sensitive to oxidative stress of WT strain. However, the Δ*dgeo*_2840*w* mutant clone showed almost identical viability as the Δ*dgeo*_2840 strain. Therefore, the non-pigment strain of WT may be malfunctioned in the carotenoid biosynthesis gene that makes the phenotype red. Δ*dgeo*_2840 strain may have a different regulatory network with more viable oxidative stress responses than WT.

### Detection of Transposition Event

**FIGURE 3:**
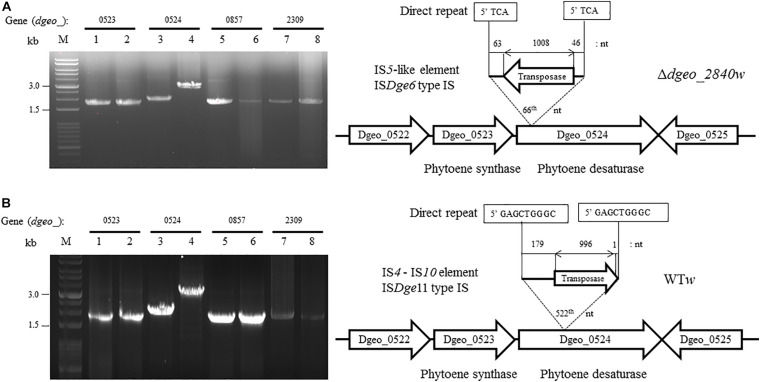
Detection of transposition event on both Δ*dgeo_*2840 mutant and WT. **(A)** Detection of IS integrated genes that contained carotenoid pathway and transposition locus on Δ*dgeo_*2840*w*. **(B)** Detection of IS integrated genes that contained carotenoid pathway and transposition locus on WT*w*. Lanes: 1, 3, 5, and 7 are parent cells; 2, 4, 6, and 8 are non-pigmented cells. Both non-pigment clones were disrupted on *dgeo*_0524 via the integration of IS*Dge6* and IS*Dge11* types on Δ*dgeo_*2840*w* and WT*w*, respectively.

To determine the transposition loci on two non-pigmented mutant strains, we performed PCR detection of the four genes related to pigment-biosynthesis: *dgeo*_0523 as phytoene synthase, *dgeo*_0524 as phytoene desaturase, and *dgeo*_0857 and *dgeo*_2309 as branched path enzymes in the carotenoid biosynthesis pathway (Lee et al., 2019). The genetic disorder of non-pigmented strains occurred by the integration of IS elements containing IS*5* family member of IS*Dge6* type for Δ*dgeo*_2840*w* mutant strain, and IS*4* family member of IS*Dge11* type for WT*w* strain. Two white strains were done by IS integration on Dgeo_0524 gene as the main key enzyme of carotenoid biosynthesis (Figure 3).

**FIGURE 4:**
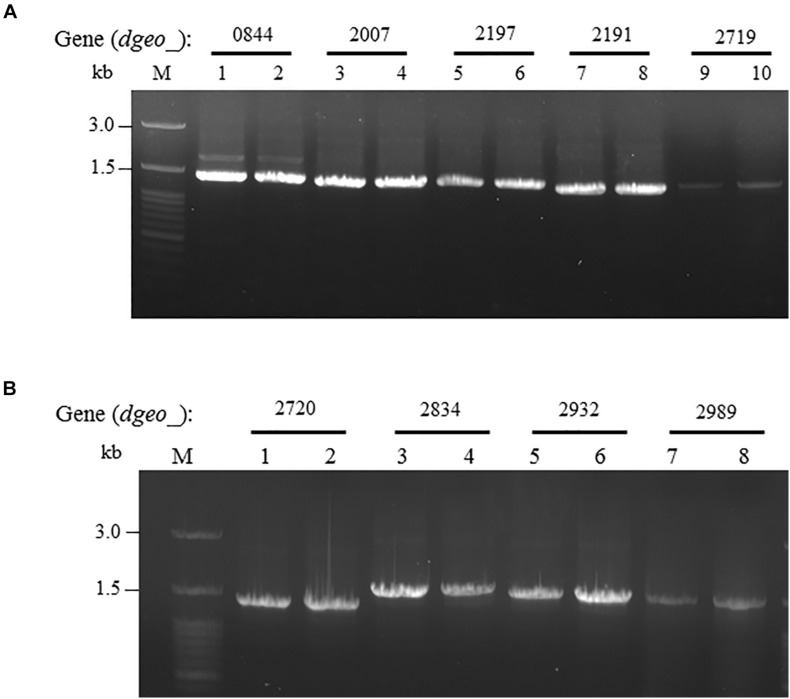
Confirming replicative type transposition of IS*Dge6* and IS*Dge11* type member. **(A)** Five IS*Dge6* type copies in Δ*dgeo_*2840 mutant strain. **(B)** Four IS*Dge11* type copies in WT. Lanes: 1, 3, 5, 7, or 9 are parent strains, while 2, 4, 6, 8, or 10 are non-pigmented strains.

IS*Dge6* type IS member has a transposase (Tnp) containing typical DDE motif, terminal inverted repeat (TIR) sequences, such as “AGACCtGCTGCGAAAcaAGGGGC,” and direct repeat (DR) sequence such as “TCA” in Δ*dgeo*_2840*w* mutant strain. This IS type has 335 aa-long Tnp with 5′ extended 46 nt length and 3′ extended 63 nt length at the end of ORF. This IS was integrated on the 66th nucleotides with counter transcriptional direction (Figure 3A). There are five copies of identical transposase gene of IS*Dge6* type on chromosome, such as Dgeo_0844, 2007, 2191, 2197, and 2719. All five IS elements are still located in their own loci and were detected by PCR using IS element region encompassing primer sets (Figure 4A and Supplementary Table S1).

The integrated IS element IS*Dge11* in WT*w* was composed of Tnp of DDE motif, TIR sequence as “CTCAGTACCTGACAGGTTGAGA,” and DR sequence as “GAGCTGGGC.” This IS type has 331 aa-long Tnp with 5′ extended 179 nt length and 3′ extended 1 nt length at the end of ORF. This IS was integrated on 522th nucleotides with the same transcriptional direction (Figure 3B). There are five identical transposase genes on genomic DNA, such as Dgeo_2720, 2834, 2932, 2989, and 2912 (Supplementary Table S2). However, *dgeo*_2912 was excluded because the 5′ end of *dgeo*_2912 was deleted 93 nt length containing the 5′ TIR sequence. The four copy genes of IS*Dge11* from genomic DNA were well-amplified with encompassing primer sets (Figure 4B). Therefore, the action mode of the transposition of IS*Dge6* and IS*Dge11* type is a replicative transposition in both non-pigmented mutant strains.

### Finding of New Transposition Loci on the Down-Regulated Genes From RNA-Seq Analysis

To define changes of gene expression by LysR family member Dgeo_2840, we performed a comparison of RNA-Seq analysis between WT strain and Δ*dgeo*_2840 mutant at optical density OD_600_ 4.0. The list of over 3.0-fold expressed genes relates to iron transporters, serine/threonine protein kinase and phosphatase, ABC transporters, and unique transposases. In particular, the up-regulated transposases belonged to 15 identical copies of IS*Dge2* type of IS*1* family IS element (Table 1). These ISs have 5′ extension of 286 nt length and 3′ extension of 11 nt length, including 12 nt TIR sequence “GGTAGTGGCTGC.” Although no transposition of IS*Dge2* type member was found, and both transposed IS*Dge6* and IS*Dge11* were not induced in Δ*dgeo*_2840 mutant strain and WT, interestingly, transposition occurred.

**TABLE 1:** Comparison of gene induction between IS*Dge2* and IS*Dge6* type transposase members on Δ*dgeo_*2840 mutant strain from RNA-Seq analysis.

**Transposase type**	**Gene**	Folds*	Loci**	**Direct repeat**	**Inverted repeat (IRL/IRR)**
IS*5* (IS*L2*)-like element IS*Dge6* type (1,122 nt)	Dgeo_0844	0.99	Chr	TCA	23 nt
	Dgeo_2007	1.09	Chr	TCA	
	Dgeo_2191	1.03	Plas1	TCA	
	Dgeo_2197	1.25	Plas1	TCA	
	Dgeo_2719	1.09	Plas1	TCA	
IS*1*-like element IS*Dge2* type (748 or 756 nt)	Dgeo_0430	8.34	Chr	–	12 nt
	Dgeo_0805	5.76	Chr	–	
	Dgeo_2533	5.88	Plas1	–	
	Dgeo_2578	9.42	Plas1	–	
	Dgeo_2595	7.64	Plas1	–	
	Dgeo_2936	6.06	Plas2	–	
	Dgeo_1673	6.85	Chr	TCTGGACA	
	Dgeo_2377	7.16	Plas1	TAGCCGCG	
	Dgeo_2436	5.79	Plas1	ATCCGGCG	
	Dgeo_2446	6.80	Plas1	GAATCCCG	
	Dgeo_2587	5.47	Plas1	AAACTCCG	
	Dgeo_2700	7.85	Plas1	CATCCATC	
	Dgeo_2795	5.28	Plas1	CAAGTTCT	
	Dgeo_2987	6.32	Plas2	CGAGTTCT	
	Dgeo_3100	7.02	Plas2	CATCTCCG	

*The fold change revealed the log2 of normalized RC ratio between WT and Δdgeo_2840 (Δdgeo_2840/WT). **Chr, chromosome; Plas1, plasmid pDGEO01; Plas2, plasmid pDGEO02.

**FIGURE 5:**
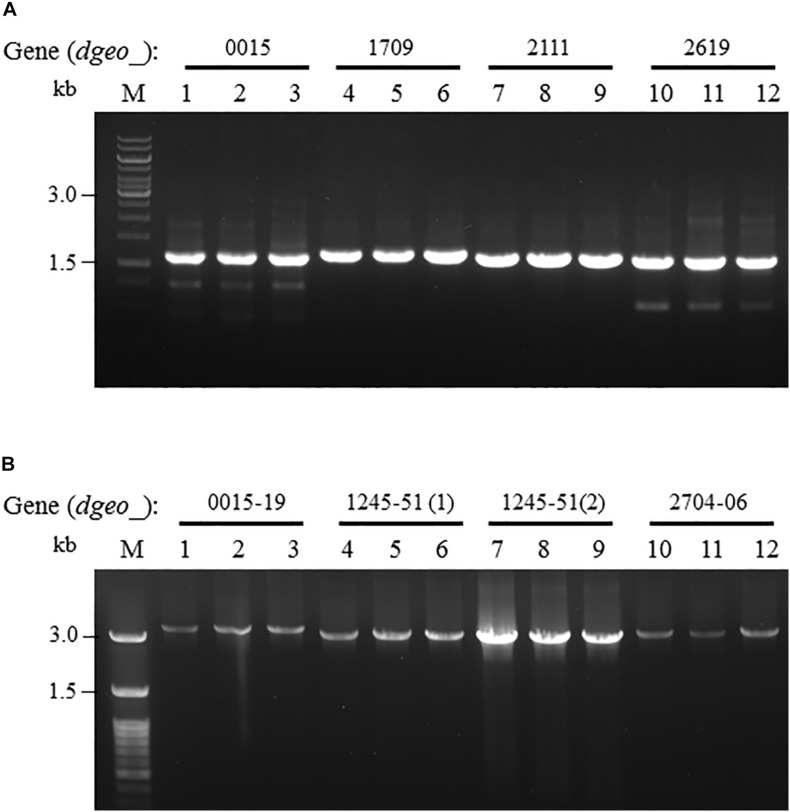
Detection of novel transposition on down-regulated genes from RNA-Seq analysis. The selected down-regulated genes by 0.2-fold from RNA-Seq analysis including **(A)** several target genes, and **(B)** extended cytochrome C or D complex gene cluster. Lines: 1, 4, 7, and 10 are WT; 2, 5, 8, and 11 are Δ*dgeo_*2840 mutant; and 3, 6, 9, and 12 are Δ*dgeo_*2840*w*.

From the RNA-Seq analysis data between Δ*dgeo*_2840 mutant and wild-type strains, we chose six down-regulated genes less than 0.3-fold, including DNA-binding regulator-cupin-chlorite dismutase (Dgeo_1708-1710), pillin (Dgeo_2111), RpiR family regulator (Dgeo_2619), and cytochrome C (Dgeo_0015-0019 and Dgeo_1247-1251) or cytochrome D complexes-thiol reductant ABC transporter subunit CydD (Dgeo_2704-2706) (Table 2 and Supplementary Table S3). Then a PCR was performed with target genes encompassing primer sets and was performed to find possible new transposition loci in Δ*dgeo*_2840, Δ*dgeo*_2840*w*, and tested with wild-type as a negative control. However, we did not find additional transposition loci (Figure 5A). In particular, we performed PCR detection of cytochrome C or D extended gene clusters, but they were not found either (Figure 5B).

**TABLE 2:** List of down-regulated cytochrome-related gene clusters on Δ*dgeo_*2840 mutant strain from RNA-Seq analysis.

**Loci**	**Gene**	Folds*	**Function**
Chromosome (Dgeo_0001 ∼ Dgeo_2347)	Dgeo_0015	0.08	Cytochrome c oxidase subunits
	Dgeo_0016	0.06	Hypothetical protein
	Dgeo_0017	0.03	Cytochrome c oxidase subunit 2
	Dgeo_0018	0.04	Cytochrome c oxidase subunit 1
	Dgeo_0019	0.23	Cytochrome c, class 1
	Dgeo_1247	0.18	Cytochrome c biogenesis protein Ccm F
	Dgeo_1248	0.18	Thiol:disulfide interchange protein
	Dgeo_1249	0.17	Cytochrome c biogenesis protein
	Dgeo_1250	0.15	Cytochrome oxidase
	Dgeo_1251	0.15	Cytochrome complex iron-sulfur subunit
Plasmid 1 (Dgeo_2348 ∼ Dgeo_2875)	Dgeo_2704	0.05	Cytochrome D ubiquinol oxidase subunit 1
	Dgeo_2705	0.06	Cytochrome D ubiquinol oxidase subunit 2
	Dgeo_2706	0.13	ABC exporter subunit CydC

*The fold change revealed the log2 of normalized RC ratio between WT and Δdgeo_2840 (Δdgeo_2840/WT).

### Induction Levels of ISs and Oxidative Stress Response Genes

**FIGURE 6:**
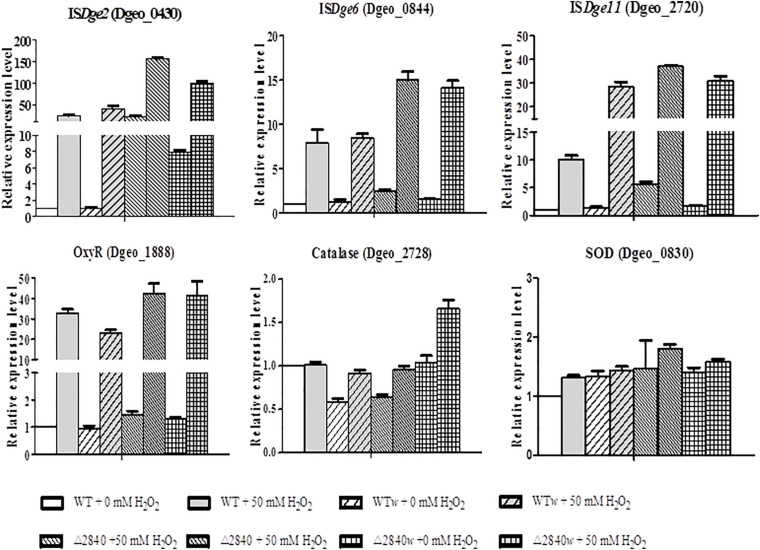
Comparison of expression levels of IS types and oxidative stress-related genes by qRT-PCR. The expression levels of three types ISs, IS*Dge2*, IS*Dge6*, and IS*Dge11*, and three oxidative stress-related genes, *oxyR*, *katE*, and *sod*, among WT and Δ*dgeo*_2840 mutant parent strains and non-pigmented mutants in the absence or presence of 50 mM H_2_O_2_. The data shown represent the means ± SDs values of three replicate experiments.

The expression levels of ISs of IS*Dge2*, IS*Dge6*, and IS*Dge11* are mainly up-regulated under oxidative stress condition (Figure 6). Both IS*Dge2* and IS*Dge11* ISs were strongly up-regulated with over 100 and 30-fold at H_2_O_2_ present condition in a LysR gene disrupted condition of Δ*dgeo*_2840 and Δ*dgeo*_2840*w*, respectively. Interestingly, IS*Dge2* was strongly up-regulated with 40 and 7.8-fold without H_2_O_2_ condition on Δ*dgeo*_2840 and Δ*dgeo*_2840*w*, respectively. These levels of RNA expression were accord well with the RNA-Seq data. IS*Dge6* was up-regulated over 14-fold on both LysR disrupted strains at H_2_O_2_ present condition. Also, WT and WT*w* showed moderate induction of three ISs at H_2_O_2_ present condition. IS*Dge11* was highly up-regulated with similar level to Δ*dgeo*_2840 mutant strains with ca 30-fold on WT*w* strain at oxidative stress condition.

All strains showed dramatic up-regulation with 30-fold of OxyR as a type of LysR (Figure 6), while other LysR members Dgeo_2711 and Dgeo_1692 were slightly up-regulated by 6 and 3-fold at H_2_O_2_ present condition, respectively (Supplementary Figure S2). However, the main ROS scavenger enzymes, catalase (Dgeo_2728) and superoxide dismutase (Dgeo_0830) were not dramatic affected by H_2_O_2_ treatment, given that among WT and Δ*dgeo*_2840 mutant strains showed less than 2-fold induction. Thus, OxyR is not a main activator for catalase, nor is it the SOD in *D. geothermalis*.

## Discussion

Active transposition of insertion sequences occurs through oxidative stress in radiation-resistant bacterium *D. geothermalis*. Using non-pigment colony selection on culture agar plate, a gene disruption of phytoene desaturase for carotenoid biosynthesis as a biomarker was successfully performed by H_2_O_2_ treatment. Here, in the absence of specialized DNA-binding protein LysR family member regulator Dgeo_2840, we first detected a non-pigmented clone, and analyzed the transposition event on a phytoene desaturase of carotenoid biosynthesis pathway. In addition, we compared wild-type non-pigmented clone that was isolated from the fifth trial of serial treatment with 50 mM H_2_O_2_. Both non-pigmented strains came through integration of a type of IS element that is IS*Dge6* and IS*Dge11* for Δ*dgeo*_2840 and WT strains, respectively. In general, these non-pigmented strains revealed more sensitivity to oxidative stress than normal reddish color strains (Lee et al., 2019). However, in the case of a less-pigmented *D. radiodurans* mutant that obtained survivors under stronger radiation, the mutant is not more sensitive to ionizing radiation than the wild-type (Okazawa and Matsuyama, 1967). Surprisingly, however, Δ*dgeo*_2840 and its non-pigment strain Δ*dgeo*_2840*w* showed high resistance to H_2_O_2_ stress, as compared with the wild-type strain (Figure 2).

### Physiological Properties of a LysR Gene Disrupted Mutant

One of the implicational reasons for the slight slower growth of the LysR family member disrupted mutant was found from the RNA-Seq results. According to those results, the expression of the three related genes of cytochrome C or D complex, *dgeo*_0015-0019, *dgeo*_1247-1251, and *dgeo*_2704-2706, was down-regulated to approximately 0.2-fold, which ultimately leads to energy generation problems (Table 2). The mutant strains were grown to the maximum optical density after an hour on TGY medium condition. Thus, the mutant strain has quickly recovered the electron transport system by undetected pathways, such as cytochrome C complex genes, *dgeo*_2837-2843, which were up-regulated by over 3-fold (data not shown).

In addition, Δ*dgeo*_2840 mutant strains were shown to be more resistant to oxidative stress than wild-type strain, but catalase (Dgeo_2728), such as a major oxidative stress protector enzyme, did not affect a gene expression level under H_2_O_2_ present condition. Because the general positive activator OxyR was known to respond immediately to oxidative stress or redox change, over 30-fold up-regulated OxyR (Dgeo_1888) was expected to be a global activator for the positive control of catalase expression in *D. geothermalis*. However, the up-regulated OxyR was not positive controlled. This phenomenon was also reported for *Corynebacterium glutaricum*. Despite *oxyR* gene being deleted, mutant strain showed higher resistance against H_2_O_2_ stress, as compared to WT strain (Milse et al., 2014). Perhaps there are different OxyR-controlling genes that may be involved in oxidative stress. Probably, Δ*dgeo*_2840 mutant also somehow has alternative responses to protect from oxidative stresses. There are additional LysR member proteins with relative low amino acid identity of maximum 34.6% (Supplementary Figure S3). The functional role of the four LysR members, including OxyR and Dgeo_2840, is still unclear about enzymes and non-enzyme defense and SOS systems in oxidation stress response.

From RNA-Seq and qRT-PCR analysis, pillin gene, *dgeo*_2111, was detected strict down-regulation with 0.08-fold (Supplementary Figure S1 and Supplementary Table S3). Pillin protein generally plays various physiological roles, such as adherence and aggregation, motility, biofilm formation, protein secretion, DNA uptake and conjugation, and electron transfer (Giltner et al., 2012). Thus, Δ*dgeo*_2840 disrupted mutant strain was found to have the potential to inhibit the transformation of foreign DNA.

### Specialized IS Induction

In *E. coli*, transposition naturally occurs in generations without stress (Sousa et al., 2018). Transposition of ISs can be induced by multiple intrinsic transpositions through host-mediated regulations such as DNA architectural proteins, DNA topologic proteins, the SOS system, and protease/chaperon system, and several extracellular triggering factors such as radiation, UV, heat shock, and some metal ions (Sawyer et al., 1987; Nagy and Chandler, 2004; Ohtsubo et al., 2005; Vandecraen et al., 2016). It may be that organisms that live in an extreme environment, such as *Deinococcus* species, have high transposition activity that leads to severe gene destruction (Pasternak et al., 2010).

From our previous data, when a DNA-binding protein Dgeo_0257, a putative Dps protein, was deleted, the IS*Dge5* and IS*Dge7* type ISs were actively transposed into other sites via replicative transposition mode. IS*Dge7* type IS was integrated into a carotenoid biosynthesis enzyme gene (Lee et al., 2019). When a member of LysR family *dgeo*_2840 was deleted, RNA-Seq results confirmed that IS*Dge2* type ISs were up-regulated, but their transposition was not detected in this study. However, in the Δ*dgeo*_2840*w* strain, an IS*Dge6* type IS was embedded into *dgeo*_0524 by replicative transposition. A non-pigmented colony was also found in the WT strain that had happened through oxidative damage. As a result, the IS*Dge11* type IS was inserted in *dgeo*_0524 gene by replicative transposition. As a result, it suggests that *dgeo*_0524 gene might be a target site of integration for several types of transposases on phenotypical reddish-colorless selection. There is a lot of demand in defining the effects of the transposition of transposable elements, especial ISs, on genomic plasticity, as well as the transposition controlling system in radiation-resistant bacteria, and their replication and transcription regulation network between a general global regulator, such as OxyR, and transposition machinery on oxidative stress. The key question is whether the transposition was caused by specialized IS elements, depending on DNA-binding proteins such as Dps and LysR, due to stronger oxidative stresses and DNA damage conditions such as γ-irradiation and plasma-radiation. It could be valuable to use advanced approaches for in-depth analysis of transposition. For examples, there are IS-Seq for genomics, which identifies new transposition loci of unique IS types, and shotgun proteomics, which identifies key player proteins such as transposases and SOS response proteins under various oxidative damage.

Phytoene desaturase (Dgeo_0524) is an enzyme that converts phytoene to lycopene. Phytoene and other carotenoids, lycopene and phytofluene, were used for skin whitening, UV blocker, and also medical needs (Rao and Agarwal, 2000; Engelmann et al., 2011; Wang, 2012). In accordance with these needs, several prokaryotic strains were applied to the production of carotenoid intermediates by target gene disruption. It has been reported that some microorganisms like *Thermococcus kodakarensis* can produce phytoene of approximately 2.6 mg/L, and 10.4 mg/L for *D. radiodurans* (Fuke et al., 2018; Jeong et al., 2018). Thus, this gene disruption approach of carotenoid gene by IS transposition event might be used for the bio-production of useful substances in the near future.

## Data Availability Statement

The original contributions presented in the study are publicly available. This data can be found here: https://www.ncbi. nlm.nih.gov/bioproject/PRJNA637617.

## Author Contributions

CL, KC, and S-JL designed the experiments, analyzed the data, and wrote the manuscript. All authors contributed to the article and approved the submitted version.

## Conflict of Interest

The authors declare that the research was conducted in the absence of any commercial or financial relationships that could be construed as a potential conflict of interest.

## Footnotes

1 www-is.biotoul.fr

2 http://www.gene-quantification.info/
