# Supplementary material for: Active Transposition of Insertion Sequences by Oxidative Stress in Deinococcus geothermalis
Source: Front Microbiol. 2020 Nov 5;11:558747. doi: 10.3389/fmicb.2020.558747 (PMC7674623; doi:10.3389/fmicb.2020.558747)
Supplement: Supplementary file 1 [file Data_Sheet_1.PDF]

Supplementary data

Lee et al. 2020

Fig. S1

A

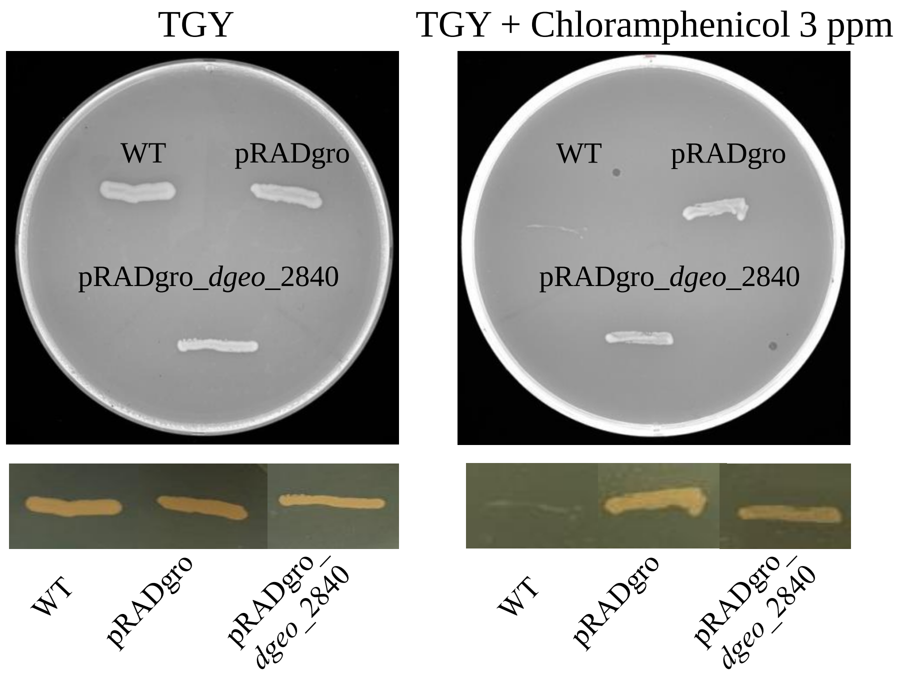

TGY
TGY + Chloramphenicol 3 ppm
WT
pRADgro
pRADgro_*dgeo*_2840
WT
pRADgro
pRADgro_*dgeo*_2840
WT
pRADgro
pRADgro_
*dgeo*_2840
WT
pRADgro
pRADgro_
*dgeo*_2840

B

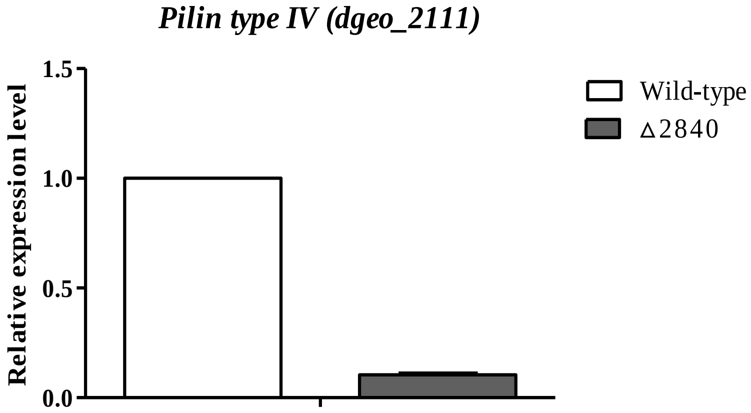

Pilin type IV (dgeo_2111)
Relative expression level
1.5
1.0
0.5
0.0
Wild-type
△2840

Fig. S2

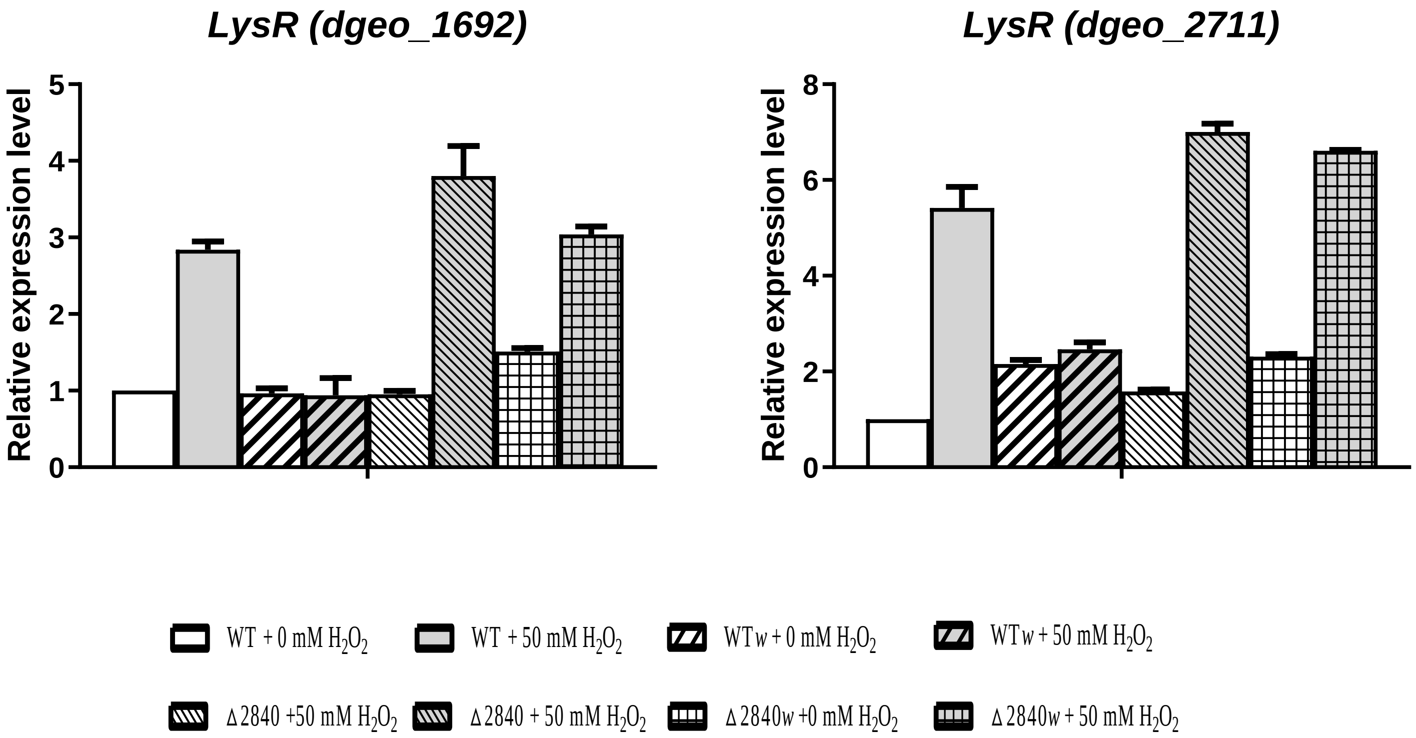

LysR (dgeo_1692)
LysR (dgeo_2711)
Relative expression level
0
1
2
3
4
5
6
8
WT + 0 mM H2O2
WT + 50 mM H2O2
WTw + 0 mM H2O2
WTw + 50 mM H2O2
Δ2840 +50 mM H2O2
Δ2840 + 50 mM H2O2
Δ2840w +0 mM H2O2
Δ2840w + 50 mM H2O2

Fig. S3

Helix-turn-Helix motif

```
Dgeo_2711       ---------------MRVNPEYLLTFSTVAEVGSVSKAAEHLHLSQPAVSGQLRALTELIG
Dgeo_2840       ----------------MLNPEHLLTFARVARLGSLSAAAEELYLTQPAVSHQLKLLTHAVG
Dgeo_1692       MADRSLLPPGQVHSPAPSLAQLRALIAVVDAGGFGEAAAELGVSQSSLSEAVARLEALAG
Dgeo_1888       -----------------MELRHLRHFVALAEEEHFGRAAERVFVVQQALSNSIKNLEDEVG
                                 .   *  :  :.    .. ** .: : * ::*  :  *    *

Dgeo_2711       EPLYTRHARGITLTEAGLELLPHAQALARTMR----QVGELAHVKRHRVKSHVRLGVSWT
Dgeo_2840       EPLFRRHRTGVHLTAAGEGLLPHAQALARALEGAQHYVQELRGLERGVLSVAASSTIAAA
Dgeo_1692       RPLLRRTPTGTLPTEAGIRALAHARAAVQAATDALLAAQEEGSLS-GTLRVASLRSTATH
Dgeo_1888       VPLVLRTTRRVQLTPAGREFLAGARETLAQAAQMVERARRAARGEVGRLTVGFVSGLAFG
                 **  *       * **   *.  *:            .  .    .   :        :

Dgeo_2711       LSPRAVTLANHFREGHPSLGIHAAHTPELIARVARGELDAALTVDASQGLPDGLEARRFA
Dgeo_2840       LLPR-VLTAFHSQYPEVTFQVRQGNTREVLDALQSGQVELALIEGPPGPLGPHLQARAFG
Dgeo_1692       LLPPALAAFRARHPGVAITVFDSEACGGGAQAVRAGRVDVGLIVS---EDATDLRLLPLP
Dgeo_1888       GLPEIVRAFRDLYP-NVSVDLRELTAQEQEAGLRGGQIDVGLMLLP--VRDPGLDSHPLW
                  *  :              .           :  *.:: .*           *    :

Dgeo_2711       SEDLRLITPAGHPLAGEGYVAPRLLAGETLLLPM--PESSVRRRAARLLEHAGVTPERPL
Dgeo_2840       EDELILVIAPTHPLAQAGLQG---VATLPLVWRE--HGSGTREVAEQALAGAGLQTRTVL
Dgeo_1692       PDEYLFVAPASR---GQHPVSCAELAAQPLILPP--QRDPCYQRVRGYLTARGVPLTQVM
Dgeo_1888       RQPLVAALPAGHALARKRRLRIGDLRDERFVFFPRHLRATYFDQVMRWCGAAGFTPNVVQ
                 :      .. :            :    ::             .       *.

Dgeo_2711       ELSSFLAVKEALVRGVGVAILPRSLVAAEVDCGLLASVGLETVE-VTLGYHAISAPL-PL
Dgeo_2840       ELPGTEAVKEAVMGGLGAAFLPERRVHREVQAGLLTRLELTLPG-LRRPLIQVTPPP-EQ
Dgeo_1692       EVEQDSVTLSMVGHGLGVTVMPQLALLPLPPGLVALPLPEPLTRPLALAVLPHRAAL-PL
Dgeo_1888       EAIEIPTLLSLVAAGIGVFLPIQFFERLSLPGVVYRPLDDAPVIEIVAVWRRDEAEGGPI
                *     .  . :  *:*. .  .          :   :       :        .

Dgeo_2711       LPGAVRTVLDRLTR-------------------------
Dgeo_2840       LSQAARTFLNLLHRQTRDQPGG-----------------
Dgeo_1692       LRAFSGVVLETVNRLKAPQGAGETLNPAPAASPLRSAPN
Dgeo_1888       VRAFLRVAQAALEKSG-----------------------
                :     .   :  :
```
