# Supplementary material for: Active Transposition of Insertion Sequences by Oxidative Stress in Deinococcus geothermalis
Source: Front Microbiol. 2020 Nov 5;11:558747. doi: 10.3389/fmicb.2020.558747 (PMC7674623; doi:10.3389/fmicb.2020.558747)
Supplement: Supplementary file 2 [file Data_Sheet_2.pdf]

Supplementary Material

Lee et al. 2020

Figure S1. Transformation of pRadgro vector into WT strain (A) and expression level of pillin by qRT-PCR (B). The empty vector and recombinant vector were transformed into WT strain. The two wild-type transformants including pRADgro or pRADgro-*dgeo*_2840 were well grown on chloramphenicol contained selection medium but WT strain without pRADgro vector not grown. The expression level of pillin (*dgeo*_2111) was strictly down-regulated with 0.1-fold on Δ*dgeo*_2840 mutant strain by qRT-PCR. This data is well matched with the RNA-Seq results in Table S3.

Figure S2. Comparison of expression levels of two LysR type genes by qRT-PCR. The expression levels of two LysR genes, *dgeo*_1692 and *dgeo*_2711, among WT and Δ*dgeo*_2840 mutant parent strains and non-pigmented mutants in the absence or presence of 50 mM H_2O_2.

Figure S3. Alignment of amino acid sequences among LysR family proteins. Four LysR family member proteins were revealed around 18.8-34.6% identity by the ClustalW (www.genome.jp/tools-bin/clustalw). The Helix-turn-Helix motif was predicted by the prabi (https://npsa-prabi.ibcp.fr).

Table S1. List of used primer sequences.

**Primer**	**Sequence (5´→3´)**	**Restriction enzyme Site**
Confirm for transposition mode		
*dgeo*_0844 _F	AGT TCC GTA AGG CGT ACA GC	-
*dgeo*_0844 _R	AAG TTT CAG CTC CTC CAG CC	-
*dgeo*_2007 _F	GCG AAC CTC AAA GAA GCC CT	-
*dgeo*_2007_R	CAG CAC GAA GGG TAC AGG C	-
*dgeo*_2191_F	CTG TGA CAG GAT CAG CCA GA	-
*dgeo*_2191_R	CGA ACT GGC GGT GGT CAA A	-
*dgeo*_2197_F	CCA GTG AGG TGG AAA TCC AC	-
*dgeo*_2197_R	CTC AGC GCG TTT CGG TTC T	-
*dgeo*_2719_F	CCT TAA CGG GCA TCG CTC C	-
*dgeo*_2719_R	CGG TGG AGA GGT CAA TGC G	-
*dgeo*_2720_F	CAA CGC ACC GAT GAA TGC C	-
*dgeo*_2720_R	TAT TTG GTC GCA GTC ACC GT	-
*dgeo*_2834_F	GCC TAT ACC CGT TCG CTC	-
*dgeo*_2834_R	TCT CCC TGT CTG GAC TGC A	-
*dgeo*_2932_F	TGA TCC CCA ACG AGA ATG GC	-
*dgeo*_2932_R	TGC CTA AAC CTC TTC CGC AG	-
*dgeo*_2989_F	TTT ATG GTT CAC CGG ACG GC	-
*dgeo*_2989_R	CAG CGT ATT GCC GAA CTG GAA	-
qRT-PCR		
*dgeo*_0430_F	GCA AGG GGG AAA CGA ACC AC	-
*dgeo*_0430_R	TCG TCG GAC TTT GAG AAG GAC A	-
*dgeo*_0844_F	GTC CAC GAT CAG GTC CTC G	-
*dgeo*_0844_R	AGA CGA GCC GCC GAA GAA G	-
*dgeo*_2720_F	GGA CCT TCC ACG AGA CGA A	-
*dgeo*_2720_R	TTC GTC CGG TCC AGC ACT A	-
*dgeo*_1133_F	CGC TCA AGA ACA TCC TCA AG	-
*dgeo*_1133_R	CCA CTC GTT GTC GTA CCA	-
*dgeo*_0830_F	TTA CCA ACT TCC CCA ACT GCC	-
*dgeo*_0830_R	TGT AGG TCT GGT GGT GCT TG	-
*dgeo*_1888_F	GAG ACG AGC GCT TTG TGT TTT	-
*dgeo*_1888_R	GTA CGA CGT TGG GCG TAA AG	-
*dgeo*_2728_F	GGA CCT TCC ACG AGA CGA A	-
*dgeo*_2728_R	TTC GTC CGG TCC AGC ACT A	-
Confirm for down-regulated gene-		
*dgeo*_0015-19_F2	CCT AGG AGA ACC TCC GTG A	-
*dgeo*_0015-19_R2	AGG TGC ACC TCC ACC CAA A	-
*dgeo*_1245-51_F1	CAG GAA AGC CAC TTG TGC C	-
*dgeo*_1245-51_R1	ACG TAG GTA TTC AGC CTC GC	-

*dgeo*_1245-51_F2	GCG AGG CTG AAT ACC TAC GT	-
*dgeo*_1245-51_R2	CGC GTA AGG GTG GAC GTA A	-
*dgeo*_2704-06_F2	CGT CGC TCT ACG CGG G	-
*dgeo*_2704-06_R2	CCT CGT GAA GTT TGG AGG CTT	-
*dgeo*_0015_F	GTT ATC TGC ATG AGG AGG TGG GC	-
*dgeo*_0015_R	TTG TCA CGG AGG TTC TCC TAG GG	-
*dgeo*_1709_F	GAC CTG ATG ATG CCC GTG ATG G	-
*dgeo*_1709_R	GCT GGA AGA TCT GGG CTG AAG AT	-
*dgeo*_2111_F	AGG TGC TAG CCA GAG CTG T	-
*dgeo*_2111_R	ATT CTT TCC TGT TTC GCC CAC C	-
*dgeo*_2619_F	AAC CGT CTA AGG AAG GCT CAA G	-
*dgeo*_2619_R	CAG CGT TCC GTC CAG ATC GA	-
Carotenoid pathway		
*dgeo*_0523_F	CTT GGC CTT GAT GAC GTT CG	-
*dgeo*_0523_R	GAG AAA GAG AGC GGT GCA GAA	-
*dgeo*_0524_F2	CGT TGA TGC GTG ATC TCT CTG	-
*dgeo*_0524_R	AGG CGG CTA TCA CGA ACT C	-
*dgeo*_0857_F	GAT GAC CCG GTT GAG TGC AT	-
*dgeo*_0857_R	CAG CGT GCG CTT TGA GAA ATG	-
*dgeo*_2309_F	GTC GCC GCC TTA TAT CCC AA	-
*dgeo*_2309_R	CAG ATG TTC GCT TTT CGC CC	-
Construction of pKRL2840 & detection		
*dgeo*_2840_F2	AAT GTA GAG CAG CGG CAA GGC	-
*dgeo*_2840_R2	AAC GCC TCC TTG ACG GCT TC	-
LF_2840	AAG GTA CCC GGG TGA ACA TCA GAT	*Kpn*1
LR_2840	AAG TCG ACC AGA ATC AAT TCG TCC T	*Sal*1
RF_2840	AAT CTA GAC TAC AGA CGA GAA CGG T	*Xba*1
RR_2840	AAC TGC AGG TCT TAA GCC TCG GA	*Pst*1

Table S2. Expression levels of five IS*Dge11* type copies from RNA-Seq analysis.

**Transposase type**	**Gene**	**Folds**	**loci**	**Direct repeat**	**Inverted Repeat (IRL/IRR)**
IS*4*(IS*10*)-like element IS*Dge11* type	*dgeo*_2720	1.21	Plas1	TCGACGAAG	22 nt
	*dgeo*_2834	1.29	Plas1	CCGCCTCAC	
	*dgeo*_2912	1.25	Plas2	-	
	*dgeo*_2932	1.50	Plas2	-	
	*dgeo*_2989	1.04	Plas2	CCTGGCAT	

Table S3. Down-regulated genes on Δ*dgeo*_2840 mutant strain from RNA-Seq analysis in this work.

**Loci**	**Gene**	**Folds**	**Function**
Chromosome	*dgeo*_1709	0.10	DNA binding regulator/ Cupin/ chlorite dismutase
Chromosome	*dgeo*_2111	0.08	pillin
Plasmid 1	*dgeo*_2619	0.28	RpiR family regulator
